# Outcomes of mitochondrial long chain fatty acid oxidation and carnitine defects from a single center metabolic genetics clinic

**DOI:** 10.1186/s13023-022-02512-5

**Published:** 2022-09-15

**Authors:** Anastasia Ambrose, Melissa Sheehan, Shalini Bahl, Taryn Athey, Shailly Ghai-Jain, Alicia Chan, Saadet Mercimek-Andrews

**Affiliations:** 1 Department of Medical Genetics, Faculty of Medicine and Dentistry, University of Alberta, 8-39 Medical Sciences Building, 8613 114 Street, Edmonton, AB T6G 2H7 Canada; 2 Princess Margaret Cancer Centre, 101 College Street, Toronto, ON M5G 1L7 Canada; 3 Department of Medical Biophysics, University of Toronto, 101 College Street, Toronto, ON M5G 1L7 Canada

**Keywords:** Mitochondrial long-chain fatty acid oxidation, Carnitine metabolism defects, Medium chain triglycerides, Long-chain fat restricted diet, Newborn screening

## Abstract

**Background:**

Mitochondrial long-chain fatty acid oxidation and carnitine metabolism defects are a group of inherited metabolic diseases. We performed a retrospective cohort study to report on the phenotypic and genotypic spectrum of mitochondrial long-chain fatty acid oxidation and carnitine metabolism defects as well as their treatment outcomes.

**Methods:**

All patients with mitochondrial long-chain fatty acid oxidation and carnitine metabolism defects were included. We divided patients into two groups to compare outcomes of those treated symptomatically (SymX) and asymptomatically (AsymX). We reviewed patient charts for clinical features, biochemical investigations, molecular genetic investigations, cardiac assessments, neuroimaging, treatments, and outcomes.

**Results:**

There were 38 patients including VLCAD (*n* = 5), LCHAD (*n* = 4), CACT (*n* = 3), MAD (*n* = 1), CPT-I (*n* = 13), CPT-II (*n* = 3) deficiencies and CTD (*n* = 9). Fourteen patients were diagnosed symptomatically (SymX), and 24 patients were diagnosed asymptomatically (AsymX). Twenty-eight variants in seven genes were identified in 36 patients (pathogenic/likely pathogenic *n* = 25; variant of unknown significance *n* = 3). Four of those variants were novel. All patients with LCHAD deficiency had the common variant (p.Glu474Gln) in *HADHA* and their phenotype was similar to the patients reported in the literature for this genotype. Only one patient with VLCAD deficiency had the common p.Val283Ala in *ACADVL.* The different genotypes in the SymX and AsymX groups for VLCAD deficiency presented with similar phenotypes. Eight patients were treated with carnitine supplementation [CTD (*n* = 6), CPT-II (*n* = 1), and MAD (*n* = 1) deficiencies]. Thirteen patients were treated with a long-chain fat restricted diet and MCT supplementation. A statistically significant association was found between rhabdomyolysis, and hypoglycemia in the SymX group compared to the AsymX group. A higher number of hospital admissions, longer duration of hospital admissions and higher CK levels were observed in the SymX group, even though the symptomatic group was only 37% of the study cohort.

**Conclusion:**

Seven different mitochondrial long-chain fatty acid oxidation and carnitine metabolism defects were present in our study cohort. In our clinic, the prevalence of mitochondrial long-chain fatty acid oxidation and carnitine defects was 4.75%.

**Supplementary Information:**

The online version contains supplementary material available at 10.1186/s13023-022-02512-5.

## Introduction

Mitochondrial long-chain fatty acid oxidation and carnitine metabolism defects are a group of inherited metabolic diseases. They are individually rare. The most commonly known mitochondrial long-chain fatty acid oxidation defects include long-chain 3-hydroxyacyl-CoA dehydrogenase (LCHAD) (encoded by *HADHA*; OMIM#600890) deficiency (OMIM#609016), very long-chain acyl-CoA dehydrogenase (VLCAD) (encoded by *ACADVL*; OMIM#609575) deficiency (OMIM#201475), carnitine transporter (encoded by *SLC22A5*; OMIM#603377) defect (CTD) (OMIM#212140), carnitine palmitoyltransferase I (CPT-I) (encoded by *CPTIA*; OMIM#600528) deficiency (OMIM#255120), carnitine palmitoyltransferase II (CPT-II) (encoded by *CPT2*; OMIM#600650) deficiency (OMIM#255110), carnitine acylcarnitine translocase (CACT) (encoded by *SLC25A20*; OMIM#613698) deficiency (OMIM#212138), and multiple acyl-CoA dehydrogenase (MAD) (encoded by *ETFDH*; OMIM#231675; *ETFA*; OMIM#608053 and *ETFB;* OMIM#130410) deficiency (OMIM#231680) [1–12].

The characteristic clinical presentations include acute hypoketotic hypoglycemia, encephalopathy, cardiomyopathy and myopathy [13, 14]. The symptoms can be precipitated by infections or prolonged fasting which results in hypoglycemia, elevated creatine kinase (CK), liver enzymes, lactate and ammonia levels. Plasma acylcarnitine profile and total and free carnitine levels can help identify specific enzyme or transporter deficiencies. The confirmation is by molecular genetic testing of candidate genes. Targeted next generation sequencing panel for myopathy and rhabdomyolysis typically includes these disorders [1, 15].

The mainstay of treatment is to prevent hypoglycemia and catabolism during intercurrent illness, vomiting or prolonged fasting which can result in death if untreated. Long-chain fat restricted diet, medium chain triglyceride (MCT), triheptanoin and carnitine supplementations are applied in mitochondrial long-chain fatty acid oxidation and carnitine metabolism defects [1, 15–21].

To report outcomes of mitochondrial long-chain fatty acid oxidation and carnitine metabolism defects, we performed a retrospective cohort study in the metabolic genetic clinic at our institution. We report on the phenotypic and genotypic spectrum of mitochondrial long-chain fatty acid oxidation and carnitine metabolism defects as well as their treatment outcomes. Furthermore, we compare the outcomes of patients identified by the manifestation of symptoms (SymX group) and asymptomatically by positive newborn screening or positive family history (AsymX group) in our study.

## Methods

The Research Ethics Office, Health Research Ethics Board, University of Alberta (Study ID: Pro00108842) approved this retrospective cohort study. All patients with the mitochondrial long-chain fatty acid oxidation and carnitine metabolism defects were included. We divided patients into two groups to compare outcomes of those treated symptomatically and asymptomatically including Group 1) SymX group: diagnosed after the onset of symptoms; Group 2) AsymX group: diagnosed by positive family history or positive newborn screening (NBS).

We reviewed patient charts for clinical features, biochemical investigations, molecular genetic investigations, cardiac assessments, neuroimaging, treatments, and outcomes. We entered all information into an Excel database (Microsoft Corp., Redmond, WA, U.S.A.).

Molecular genetic investigations using patient and parent DNA samples were performed in clinical molecular genetic laboratories according to their methods. We applied American College of Medical Genetics and Genomics and the Association for Molecular Pathology (ACMG/AMP) variant classification guidelines for interpretation of variants [22]. We also searched all variants in the Genome Aggregation Database (gnomAD) (http://gnomad.broadinstitute.org/about) for their allele frequency in the general population [23].

We performed statistical analysis using R statistical software (version 4.0.2). Results are given as mean ± SD (range). Non-parametric Fisher’s exact and Wilcoxon rank-sum tests were chosen to compare outcomes between groups as indicated where appropriate. Results were considered statistically significant with a *p*-value of < 0.05.

## Results

There were 38 patients (16 males, 22 females) from 27 unrelated families diagnosed with mitochondrial long-chain fatty acid oxidation and carnitine metabolism defects. Their current average age was 15.3 ± 16.9 standard deviation (SD) years (range 3 months-55 years). There were 15 adults (> 18 years of age) (average age of 36.5 ± 11.2 SD years and age range of 22–55 years) and 23 children (average age of 4.5 ± 3.1 SD years and range of 3 months-12 years). The mitochondrial long-chain fatty acid oxidation and carnitine metabolism defects diagnosed in our patient cohort were VLCAD (*n* = 5), LCHAD (*n* = 4), CACT (*n* = 3), MAD (*n* = 1), CPT-I (*n* = 13), CPT-II (*n* = 3) deficiencies and CTD (*n* = 9). The CTD, LCHAD and VLCAD deficiencies are included in the newborn screening programs in our Province of Alberta.

Fourteen patients were diagnosed symptomatically and were included in the SymX group. Their average age was 21.4 ± 18.3 SD years (range 7 months-55 years). Twenty-four patients were included in the AsymX group and were diagnosed either by positive NBS (*n* = 9) (average age of 5.8 ± 3.4 SD years and range of 3 months-12 years) or by positive family history (*n* = 15) (average age of 21 ± 19.1 SD years and range of 5 months-53 years). We summarized the type of mitochondrial long-chain fatty acid oxidation and carnitine metabolism defects, clinical features, biochemical features, genotypes, cardiac investigations, and treatments of patients in the SymX group in Table 1 and in the AsymX group in Table 2. Additionally, NBS results of the AsymX group are listed in Additional file1: Table S1.

**Table 1:** Clinical, biochemical and molecular genetic features of SymX patients are summarized in Table 1

Patient#/study ID/sex/diagnosis/age of diagnosis/current age	Presenting symptom (age of onset)/other clinical features	Initial investigations AC^1,2,3^/TC^1,2,3^/FC^1,2,3^/UOA	Molecular genetic investigations	Outcome
1/1/F/CPT-I deficiency/2yrs/26yrs	Seizure (2yrs)/hypoglycemia, GTCS	N/N/N/↑AA, SUA, 3OH-SEA/NP	HMZ c.1436C > T (p.Pro479Leu) in *CPTIA*	Hospital admissions (*n* = 5), normal echo (26yrs)
2/3/F/CPT- I deficiency /neonatal/3yrs	Hypoglycemia (neonatal)/no	N/N/N/↑2-oxoadipic acid, 4-HPPA, 4-HPLA/NP	HMZ c.1436C > T (p.Pro479Leu) in *CPTIA*	Normal
3/7/F/CPT- I deficiency /2mo/3yrs	Hepatosplenomegaly (2mo)/no	N/NP/62.01/N/NP	HMZ c.1436C > T (p.Pro479Leu) in *CPTIA*	Normal echo (3yrs)
4/10/F/CPT- I deficiency/neonatal/3yrs	Hypoglycemia (neonatal)/no	NP	HMZ c.1436C > T (p.Pro479leu) in *CPTIA*	Hospital admissions (*n* = 1)
5/49/F/CPT- I deficiency/5yrs/22yrs	RM (5yrs)/hypoglycemia	N/NP/18.5/NP/NP	HMZ c.1436C > T (p.Pro479Leu) in *CPTIA*	Hospital admissions (*n* = 2)
6/6/M/CPT-II deficiency/35yrs/51yrs	Myopathy (18yrs)/ RM, ATN, fatigue, myalgia, myoglobinuria	N/NP/NP/↑lactic acid/NP	NP	Hospital admissions (*n* = 3)
7/14/F/CPT-II deficiency/15yrs/24yrs	RM (15yrs)/myalgia	C18:1OH = 0.05/N/N/NP/NP	CMP HTZ c.298delG (p.Val100Leufs*30)/c.338C > T (p.Ser113Leu) in *CPT2*	Hospital admissions (*n* = 12)
8/15/F/CPT-II deficiency/35yrs/36yrs	RM (20yrs)/myoglobinuria, myopathy, ATN	C16 = 0.74, C18:1 = 0.85, C18:2 = 0.51/N/N/NP/NP	CMP HTZ c.341-2621_1121del/c.338C > T (p.Ser113Leu) in *CPT2*	Hospital admissions (*n* = 2), normal echo (35yrs)
9/37/M/LCHAD deficiency/47yrs/55yrs	RM (4yrs)/retinopathy, myopathy, peripheral neuropathy	C16:1OH = 0.08, C16OH = 0.18, C18:1OH = 0.16, C18OH = 0.16/N/N/NP/NP	HTZ c.1528G > C (p.Glu474Gln) in *HADHA*	Hospital admissions (*n* = 16), normal echo (53yrs)
10/42/M/VLCAD deficiency/10mo/25yrs	Hypoglycemia (10mo)/DCMP, myalgia, RM, myopathy, hypoglycemia	C14:1 = 4.15, C14:2 = 0.46/78/57/N/NP	CMP HTZ c.605 T > A (p.Leu202His) /c.1182 + 1G > A in *ACADVL*	Hospital admissions (*n* = 3), normal echo (23yrs)
11/52/F/VLCAD deficiency/neonatal/7mo	Hypoglycemia (neonatal)/hypotonia	C14:2 = 0.94, C14:1 = 9.03/119.9/32.6/↑AA, SUA, SEA/NP	CMP HTZ c.1182 + 1G > A/ c.1406G > A (p.Arg469Gln) in *ACADVL*	Hospital admissions (*n* = 4), normal echo (2wks)
12/45/M/CACT deficiency/neonatal/3yrs	Hypoglycemia (neonatal)/no	C16 = 1.36, C18:1 = 0.70, C16-DC = 0.15 /N/N/NP/NP	CMP HTZ c.897dupC (p.Asn300Glnfs*24)/c.269 T > G (p.Phe90Cys) in *SLC25A20*	Normal echo (3yrs)
13/47/M/CACT deficiency/neonatal/deceased	Hypotonia, cardiac arrest, lactic acidemia, hypoglycemia (neonatal)/no	C16 = 13.66, C18:1 = 4.31/N/20.51/NP/NP	CMP HTZ c.326 + 1delG (IVS3 + 1delG) /c.10C > T (p.Gln4X) in *SLC25A20*	Hospital admissions (*n* = 1), left ventricular dysfunction, MV, TV tricuspid regurgitation in echo (5 d)
14/48/M/MAD deficiency/4yrs/26yrs	Lethargy (8mo)/seizure, hypoglycemia	Elevated C4-C14^a^/106/40/↑AA/NP	CMP HTZ c.524G > A (p.Arg175His) /c.1001 T > C (p.Leu334Pro) in *ETFDH*	Hospital admissions (n = 3), normal echo (24yrs)

*Reference Ranges:* Free carnitine^1^ 7.3–30.4; Free carnitine^2^ 18.6–55.0; Free carnitine^3^ 25.3–57.0; Total carnitine^1^ 14.0–47.0; Total carnitine^2^ 24.9–72.1; Total carnitine^3^ 32.5–73.6; C16^1^ 0.04–0.41; C16^2^ 0.03–0.22; C16^3^ 0.03–0.19; C16-DC^1^ < 0.06; C16-DC^2^ < 0.03; C16-DC^3^ 0.03–0.09; C16-OH^1^ < 0.05; C16-OH^2^ < 0.04; C16-OH^3^ < 0.02; C18:1^1^ 0.04–0.20; C18:1^2^ 0.03–0.23; C18:1^3^ 0.03–0.26; C18:2^1^ < 0.10; C18:2^2^ < 0.12; C18:2^3^ < 0.13; C18-OH^1^ < 0.03; C18-OH^2^ < 0.03; C18-OH^3^ < 0.02; C18:1OH^1^ < 0.02; C18:1OH^2^ < 0.03; C18:1OH^3^ < 0.02; C16:1OH^1^ < 0.27; C16:1OH^2^ < 0.11; C16:1OH^3^ < 0.03; C14:1^1^ < 0.26; C14:1^2^ < 0.28; C14:1^3^ < 0.11; C14:2^1^ < 0.10; C14:2^2^ < 0.13; C14:2^3^ < 0.11

^1^ Indicates reference range for age group newborn-2 months

^2^ Indicates reference ranges for age group 2 months-18 years

^3^ Indicates reference ranges for age group 18 years and up

*4-HPPA* 4-OH-phenylpyruvic acid; *4-HPLA* 4-hydroxyphenyllactic acid; *AA* adipic acid; *AC* acylcarnitines; *ATN* acute tubular necrosis; *CACT* carnitine acylcarnitine translocase; *Car* carnitine; *CMP HTZ* compound heterozygous; *CPT-I* carnitine palmitoyltransferase I; *CPT-II* carnitine palmitoyltransferase II; *CTD* carnitine transporter defect; *DCMP* dilated cardiomyopathy; echo echocardiography; *FC* free carnitine; F female; *HMZ* homozygous; *LCHAD* long-chain 3-hydroxyacyl-CoA dehydrogenase; *MAD* multiple acyl-CoA dehydrogenase; mo months; *MV* mitral valve; *NB* newborn; *N* normal; *NP* not performed; *NR* not reported; RM rhabdomyolysis; *SEA* sebacic acid; *SUA* suberic acid; *TV* tricuspid valve; *UOA* urine organic acids; yrs year(s); *VLCAD* very long-chain acyl-CoA dehydrogenase

^a^Result report did not provide values

**Table 2:** Clinical, biochemical and molecular genetic features of AsymX patients are summarized in Table 2

Patient #/study ID/sex/diagnosis/age of diagnosis/current age	Presenting symptom (age of onset)/other clinical features	Initial investigations AC^1,2,3^/TC^1,2,3^/FC^1,2,3^/UOA/TRR%	Molecular genetic investigations	Outcome
1/2/M/CPT-I deficiency/neonatal/3yrs	No	N/NP/75.4/↑3-HIVA /NP	HMZ c.1436C > T (p.Pro479Leu) in *CPTIA*	Normal
2/5/F/CPT-I deficiency/6yrs/26yrs	No	NP	HMZ c.1436C > T (p.Pro479Leu) in *CPTIA*	Normal
3/8/M/CPT-I deficiency/45yrs/53yrs	No	NP	HMZ c.1436C > T (p.Pro479Leu) in *CPTIA*	Normal
4/9/F/CPT-I deficiency/neonatal/6yrs	No	N/N/117.75/NP/NP	HMZ c.1436C > T (p.Pro479Leu) in *CPTIA*	Normal echo (4yrs)
5/11/F/CPT-I deficiency/39yrs/48yrs	No	NP	HMZ c.1436C > T (p.Pro479Leu) in *CPTIA*	Normal
6/12/F/CPT-I deficiency/neonatal/2yrs	No	N/66.9/51.3/N/NP	HMZ c.1436C > T (p.Pro479Leu) in *CPTIA*	atrial septal aneurysm, PFO, ASD, PDA (1mo)
7/50/F/CPT-I deficiency/2yrs/3yrs	No	NP	HMZ c.1436C > T (p.Pro479Leu) in *CPTIA*	Normal
8/51/M/CPT-I deficiency/neonatal/5yrs	No	NP	HMZ c.1436C > T (p.Pro479Leu) in *CPTIA*	Normal
9/18/M/CTD deficiency/neonatal/7yrs	No	NP/NP/NP/NP/NP	HMZ c.769C > T (p.Arg257Trp) in *SLC22A5*	Normal echo (5yrs)
10/19/F/CTD deficiency/32yrs/37yrs	No	NP/2.2/1.7/N/90.88	HMZ c.845G > A (p.Arg282Gln) in *SLC22A5*	Normal
11/24/M/CTD deficiency/neonatal/3yrs	No	N/6.4/4.2/N/NP	HMZ c.845G > A (p.Arg282Gln) in *SLC22A5*	Hospital admissions (*n* = 2), normal echo (2yrs)
12/25/M/CTD deficiency/neonatal/3mo	No	NP/6.1/4.8/N/NP	HMZ c.845G > A (p.Arg282Gln) in *SLC22A5*	Normal
13/22/F/CTD deficiency/38yrs/44yrs	No/ fatigue (retrospectively at the time of diagnosis)	NP/ < 2/ < 2/NP/NP	HMZ c.248G > T (p.Arg83Leu) in *SLC22A5*	Normal echo (42yrs)
14/23/F/CTD deficiency/28yrs/40yrs	No/ fatigue (retrospectively at the time of diagnosis)	NP/ < 2/ < 2/NP/NP	NP	Normal echo (30yrs)
15/28/M/CTD deficiency/neonatal/6yrs	No	N/10.6/8.4/NP/NP	CMP HTZ c.1463G > A (p.Arg488His)/c.424G > T (p.Ala142Ser)/c.136C > T (p.Pro46Ser) in *SLC22A5*	Normal echo (5yrs)
16/32/F/CTD deficiency/26yrs/33yrs	No/ fatigue (retrospectively at the time of diagnosis)	NP/10.1/5.1/N/NP	CMP HTZ c.364G > T (p.Asp122Tyr)/ c.505C > T (p.Arg169Trp) in *SLC22A5*	Normal echo (27yrs)
17/36/M/CTD deficiency/neonatal/9yrs	No/ myalgia (8yrs)	NP/NP/NP/NP/NP	CMP HTZ c.424G > T (p.Ala142Ser) /c.1463G > A (p.Arg488His) /c.1324GC_1325AT (p.Ala442Ile) in *SLC22A5*	Normal echo (6yrs)
18/38/M/LCHAD deficiency/neonatal/6yrs	No/ RM (2yrs), retinopathy, myopathy (6 yrs)	C16OH = 0.73, C18OH = 0.87, C18:1OH = 0.54/N/N/N/NP	HMZ c.1528G > C (p.Glu474Gln) in *HADHA*	Hospital admissions (*n* = 6), normal echo (6yrs)
19/39/F/LCHAD deficiency/neonatal/3yrs	No/RM, myopathy	C16OH = 1.05, C18OH = 0.91, C18:1OH = 1.1/N/NP/NP/NP	HMZc.1528G > C (p.Glu474Gln) in *HADHA*	Hospital admissions (*n* = 4), normal echo (3yrs)
20/53/M/LCHAD deficiency/neonatal/7mo	Hypoglycemia (neonatal, but diagnosed prenatally)	C16:1OH = 0.33, C16OH = 0.78, C18:1OH = 0.45, C18OH = 0.40/N/N/N/NP	HMZ c.1528G > C (p.Glu474Gln) in *HADHA*	Hospital admissions (*n* = 2), normal echo (12yrs)
21/40/F/VLCAD deficiency/neonatal/7yrs	No/ RM, myopathy (3 yrs)	C14:1 = 0.13, C14:2 = 0.06/N/N/NP/NP	CMP HTZ c.1375dupC (p. p.Arg459ProfsX4)/c.1700G > A (p.Arg567Gln) in *ACADVL*	Hospital admissions (n = 1), dilated ascending aorta in echo (6yrs)
22/43/F/VLCAD deficiency/neonatal/12yrs	No/RM, myalgia (12 yrs)	N/N/N/↑AA, SUA, SEA/NP	CMP HTZ c.848 T > C (p.Val283Ala)/ c.1375dupC (p.Arg459ProfsX4) in *ACADVL*	Hospital admissions (*n* = 1)
23/44/F/VLCAD deficiency/neonatal/9yrs	No/ myalgia (4yrs)	N/N/N/NP/NP	CMP HTZ c.779C > T (p.Thr260Met/ c.1733 T > C (p.Met578Thr) in *ACADVL*	Hospital admissions (*n* = 3), normal echo (7yrs)
24/46/F/CACT deficiency/4yrs/8yrs	No	C16 = 0.85, C18:1 = 0.83, C18:2 = 0.25/N/ 14.9/NP/NP	CMP HTZ c.897dupC (p.Asn300Glnfs*24)/c.269 T > G (p.Phe90Cys) in *SLC25A20*	PDA in echo (6mo)

*Reference Ranges:* Free carnitine^1^ 7.3–30.4; Free carnitine^2^ 18.6–55.0; Free carnitine^3^ 25.3–57.0; Total carnitine^1^ 14.0–47.0; Total carnitine^2^ 24.9–72.1; Total carnitine^3^ 32.5–73.6; C16^1^ 0.04–0.41; C16^2^ 0.03–0.22; C16^3^ 0.03–0.19; C16-DC^1^ < 0.06; C16-DC^2^ < 0.03; C16-DC^3^ 0.03–0.09; C16-OH^1^ < 0.05; C16-OH^2^ < 0.04; C16-OH^3^ < 0.02; C18:1^1^ 0.04–0.20; C18:1^2^ 0.03–0.23; C18:1^3^ 0.03–0.26; C18:2^1^ < 0.10; C18:2^2^ < 0.12; C18:2^3^ < 0.13; C18-OH^1^ < 0.03; C18-OH^2^ < 0.03; C18-OH^3^ < 0.02; C18:1OH^1^ < 0.02; C18:1OH^2^ < 0.03; C18:1OH^3^ < 0.02; C16:1OH^1^ < 0.27; C16:1OH^2^ < 0.11; C16:1OH^3^ < 0.03; C14:1^1^ < 0.26; C14:1^2^ < 0.28; C14:1^3^ < 0.11; C14:2^1^ < 0.10; C14:2^2^ < 0.13; C14:2^3^ < 0.11

^1^ Indicates reference range for age group newborn-2 months

^2^ Indicates reference ranges for age group 2 months-6 years

^3^ Indicates reference ranges for age group 18 years and up

*3-HIVA* 3-OH-isovaleric acid; *AA* adipic acid; *AC* acylcarnitines; *ASD* atrial septal defect; *CACT* carnitine acylcarnitine translocase; *Car* carnitine; *CPT-I* carnitine palmitoyltransferase I; *CPT-II* carnitine palmitoyltransferase II; *CMP HTZ* compound heterozygous; *CTD* carnitine transporter defect; *Echo* echocardiography; *FC* free carnitine; *F* female; *HMZ* homozygous; *LCHAD* long-chain 3-hydroxyacyl-CoA dehydrogenase; *MAD* multiple acyl-CoA dehydrogenase; *mo* months; *NB* newborn; *N* normal; *NP* not performed; *NR* not reported; *PDA* patent ductus arteriosus; *PFO* patent foramen ovale; *RM* rhabdomyolysis; *SEA* sebacic acid; *SUA* suberic acid; *TRR* tubular reabsorption rate; *UOA* urine organic acids; *yrs* year(s); *VLCAD* very long-chain acyl-CoA dehydrogenase

Thirty-six patients had molecular genetic confirmation of their specific mitochondrial long-chain fatty acid oxidation and carnitine metabolism defects. All variants identified in our study cohort were re-classified using ACMG/AMP criteria (Additional file 1: Table S2). Unfortunately, two patients (CPT-II *n* = 1; CTD *n* = 1) did not have molecular genetic investigations due to the limited publicly available funding for molecular genetic investigations at the time of their diagnosis. In one patient with LCHAD deficiency, a single variant was identified. There were 28 different variants from 36 patients including 25 pathogenic or likely pathogenic variants and three variants of unknown significance. The most common variant type was missense variant (*n* = 20). Fourteen patients had compound heterozygous variants and 21 patients had homozygous variants (Tables 1 and 2). Four of the variants were not reported in the literature previously including *CPT2* (*n* = 2), and *SLC25A20* (*n* = 2).

Eight patients were treated with carnitine supplementation [CTD (*n* = 6), CPT-II (*n* = 1), and MAD (*n* = 1) deficiencies]. The average carnitine dose was 48.6 mg/kg/day (range 6.1–125 mg/kg/day). Thirteen patients were treated with a long-chain fat restricted diet and MCT supplementation (Table 3). Diet therapy recommendations were individualized based on the type of mitochondrial long-chain fatty acid oxidation and carnitine metabolism defects as well as clinical judgement regarding disease severity. The most severe patients were prescribed the greatest long chain fat restrictions and shortest fasting allowances. Recommendations for long chain fat restriction ranged from 5% of total energy for severe phenotypes to 35% of total energy for mild or moderate phenotypes. MCT was supplemented as an alternative energy source to meet total fat requirements, providing 0 to 35% of total energy. MCT supplementation was provided using either medical formulas (Lipistart) or module forms (MCT oil, MCT procal, Betaquick, or Liquigen). Age dependent maximum hours of fasting recommendations were applied. The longest allowed overnight fasting was up to 12 h in older children and adults. For illness management, carbohydrate was provided either through glucose polymers (such as Solcarb) or IV glucose, depending on the patient’s ability to tolerate feeding (and thus stay at home) versus require admission. MCT supplementation was also used in some cases as an alternative energy source to long-chain fat. Details of complex carbohydrate and/or MCT intakes prior to exercise as well as the compliance and moderation in exercise level are summarized in Additional file 1: Table S3.

**Table 3:** All SymX and AsymX patients on the diet are summarized in Table 3

Groups	Patient#/study ID/diagnosis	Long chain fat intake		MCT		Protein Intake (g/kg/d)
		Recommended intake	Actual intake	Recommended intake	Actual intake	
SymX group	6/6/CPTII def	20%	30%	NA	NA	1.09
	7/14/CPTII def	20%	< 20%	10–15%	1%	1.17
	8/15/CPTII def	35%	44%	10–20%	10–20%	1.33
	9/37/LCHAD def	10%	8%	10–15%	0%	NA
	10/42/VLCAD def	10–15%	6%	10–15%	21%	2.12
	11/52/VLCAD def	5%	5%	35%	35%	2.94
	14/48/MAD def	25–30%	33%	NA	NA	2.32
AsymX group	24/38/LCHAD def	10%	7%	15–25%	23%	2.57
	25/39/LCHAD def	10–15%	10%	20–25%	22%	2.13
	26/53/LCHAD def	10%	10%	30%	32%	2.30
	27/40/VCLAD def	15–25%	26%	5%	0%	0.97
	28/43/VLCAD def	15–25%	13%	13%	13%	1.51
	29/44/VLCAD def	10–20%	10–12%	15–20%	15%	2.15

*CACT* carnitine acylcarnitine translocase; *CPT-I* carnitine palmitoyltransferase I; *CPT-II* carnitine palmitoyltransferase II; *CTD* carnitine transporter defect; *def* deficiency; *LCHAD* long-chain 3-hydroxyacyl-CoA dehydrogenase; *MAD* multiple acyl-CoA dehydrogenase; *VLCAD* very long-chain acyl-CoA dehydrogenase

### Clinical and biochemical features and treatment outcomes of patients in the SymX group (*n* = 14)

There were 14 patients with CPT-I (*n* = 5), CPT-II (*n* = 3), LCHAD (*n* = 1), VLCAD (*n* = 2), CACT (*n* = 2) and MAD (*n* = 1) deficiencies. The average current age was 21.4 ± 18.3 SD years (range 7 months-55 years). The average age of symptom onset was 4.5 ± 7.4 SD years (range newborn-23 years). The average age of diagnosis was 10.6 ± 16.1 SD years (range newborn-47 years). Two patients with CPT-I deficiency presented at birth with hypoglycemia and two patients with CACT deficiency presented with symptoms prior to receiving NBS results. The average lack of time between the symptom onset and diagnosis was 6.1 ± 12.1 SD years (range newborn-43 years). The average duration of follow-up was 10.6 ± 8.8 years SD (range 7 months-24.2 years). The symptom frequency for each disorder is summarized in Table 4.

**Table 4:** Frequency of clinical features of the SymX and AsymX patients are summarized in Table 4

Features	Abnormal clinical, biochemical and organ involvement	SymX group (n = 14)	AsymX group (NBS or asymptomatic with positive family history (*n* = 21)	Statistical analysis (Fisher’s Exact Test)
Clinical features	Seizure	14.3% (n = 2)	0%	0.1529
	Myalgia	35.7% (*n* = 5)	9.5% (*n* = 2)	0.0897
	Fatigue	14.3% (*n* = 2)	0%	0.1529
	Lethargy	14.3% (*n* = 2)	4.8% (*n* = 1)	0.5508
	Hepatomegaly	14.3% (*n* = 2)	0%	0.1529
	Hypotonia	7.1% (*n* = 1)	4.8% (*n* = 1)	1
	Headache	0%	0%	1
	Peripheral neuropathy	7.1% (*n* = 1)	0%	0.4000
	Retinopathy/maculopathy	7.1% (*n* = 1)	4.8% (*n* = 1)	1
Investigations	Rhabdomyolysis	50% (*n* = 7)	9.5% (*n* = 2)	0.0153*
	Lactic acidemia	14.3% (*n* = 2)	0%	0.1529
	Myopathy	14.3% (*n* = 2)	0%	0.1529
	Cardiac arrhythmia	7.1% (*n* = 1)	0%	0.4000
	Dilated cardiomyopathy	7.1% (*n* = 1)	0%	0.4000
	Acute tubular necrosis (acute kidney insufficiency)	14.3% (*n* = 2)	0%	0.1529
	Hypoglycemia	71.4% (*n* = 10)	4.8% (*n* = 1)	5.126 × 10^–5^*
	Myoglobinuria	21.4% (*n* = 3)	0%	0.0556
Hospital admissions^a^	CTD	No patients in this group with CTD	0% (*n* = 0 out of 6)	1
	CPT-I deficiency	40% (*n* = 2 out of 5)	0% (*n* = 0 out of 8)	0.1282
	CACT deficiency	50% (*n* = 1 out of 2)	100% (*n* = 1 out of 1)	1
	LCHAD deficiency	100% (*n* = 1 out of 1)	100% (*n* = 3 out of 3)	1
	VLCAD deficiency	100% (*n* = 2 out of 2)	100% (*n* = 3 out of 3)	1
Treatment^a^	Carnitine	14.3% (*n* = 2 out of 14)	28.6% (*n* = 6 out of 21)	0.4307
	Long-chain fat restriction	50% (*n* = 7 out of 14)	28.6% (*n* = 6 out of 21)	0.2882

*CACT* carnitine acylcarnitine translocase; *CPT-I* carnitine palmitoyltransferase I; *CPT-II* carnitine palmitoyltransferase II; *CTD* carnitine transporter defect; *LCHAD* long-chain 3-hydroxyacyl-CoA dehydrogenase; *MAD* multiple acyl-CoA dehydrogenase; *VLCAD* very long-chain acyl-CoA dehydrogenase

^*^Significant *p*-value (< 0.05)

^a^Values are reported as averages. Long-chain fat restriction and MCT supplementation indicate percentage of daily intake

The most common features were hypoglycemia (*n* = 10) and rhabdomyolysis (*n* = 7). Patients with CPT-I deficiency had hypoglycemia (*n* = 4), seizure (*n* = 1), rhabdomyolysis (*n* = 1) and hepatomegaly (*n* = 2). Patients with CPT-II deficiency had rhabdomyolysis (*n* = 3), myoglobinuria (*n* = 2), myalgia (*n* = 3), acute tubular necrosis (*n* = 2), hypoglycemia (*n* = 1) and fatigue (*n* = 1). One patient with LCHAD deficiency had retinopathy, rhabdomyolysis, fatigue, myalgia, myopathy, myoglobinuria, and peripheral neuropathy (symptoms of loss of deep tendon reflexes and pinprick and light touch sensation in lower extremities). Patients with VLCAD deficiency had hypoglycemia (*n* = 2), rhabdomyolysis (*n* = 2), cardiomyopathy (*n* = 1), myalgia (*n* = 1), myopathy (n = 1), lethargy (*n* = 1), hypotonia (*n* = 1), and lactic acidemia (*n* = 1). Patients with CACT deficiency had hypoglycemia (*n* = 2), hypotonia (*n* = 1), and lactic acidemia (*n* = 1). One patient with MAD deficiency had hypoglycemia, seizure, and lethargy. Interestingly one patient with VLCAD deficiency presented with neonatal hypoglycemia and was admitted to neonatal intensive care unit prior to NBS results becoming available.

Nine patients had alanine aminotransferase (ALT) (*n* = 9) and/or aspartate aminotransferase (AST) (*n* = 8) during regular follow-up appointments. Six patients (CPT-I *n* = 1; CPT-II *n* = 3; VLCAD *n* = 1 and CACT *n* = 1 deficiencies) had normal ALT levels. Six patients (CPT-I *n* = 2; CPT-II *n* = 2; VLCAD *n* = 1 and CACT *n* = 1 deficiencies) had normal AST levels.

Seven patients [(CPT-II (*n* = 3), LCHAD (*n* = 1), VLCAD (*n* = 2), MADD (*n* = 1) deficiencies] were treated with a long-chain fat restricted diet. The average age of diet initiation was 20.4 ± 18.2 SD years (range newborn-48 years). The average recommended long-chain fat intake was 19% ± 0.10 SD of total daily intake (range 5–35%), whereas the average of actual long-chain fat intake was 24% ± 0.1 SD of total daily intake (range 5–44%). The average recommended MCT intake was 16% ± 0.1 SD of daily total intake (range 0–35%), whereas the average actual MCT intake was 13% ± 0.1 SD of total daily intake (range 0–21%). We recommended pre-exercise complex carbohydrates in two patients (CPT-II *n* = 1; and MAD *n* = 1 deficiencies) to prevent rhabdomyolysis associated with exercise. Additionally, we recommended pre-exercise complex carbohydrates and MCT oil (in any form) in four patients (VLCAD *n* = 1; LCHAD *n* = 1; and CPT-II *n* = 2 deficiencies) to prevent rhabdomyolysis associated with exercise (Additional file 1: Table S3). Three patients were compliant with these recommendations including patients with CPT-II (*n* = 1), VLCAD (*n* = 1) and MAD (*n* = 1) deficiencies. Three patients were not compliant with our recommendations and for two of them with CPT-II deficiency we recommended limited level of exercise to prevent repeated rhabdomyolysis episodes. One non-compliant patient with LCHAD deficiency did not exercise. None of the patients received additional oil or cornstarch supplementation at the last assessment. One patient with CPT-II deficiency was non-compliant since the diagnosis (at age 9 years) due to very high exercise levels and failure to consume an appropriate amount of carbohydrates leading to several hospital admissions for the treatment of rhabdomyolysis. One patient with LCHAD deficiency had difficulty in being compliant with the long-chain fat restricted diet leading to multiple hospital admissions for rhabdomyolysis since the diagnosis. The adherence to the diet was improved with the use of nasogastric feeds, but this was not sustainable for long-term.

Adherence to diet recommendations was suboptimal in the SymX group. 50% (7 out of 14) of the patients were prescribed diet therapy including long chain fat restriction and, in some cases, MCT supplementation. 43% (3 out of 7) of the patients met their long chain fat goals within 1% of max recommendations or took less long chain fat than prescribed. Those that did not meet their long chain fat goals were not monitoring daily fat intake so had day-to-day variability in fat consumption. 71% (5 out of 7) of the patients were prescribed MCT supplements, where 60% (3 out of 5) of the patients met their goals. Two patients, who did not meet MCT goals, struggled with gastrointestinal side effects. One patient experienced significant abdominal discomfort and bloating. Another patient’s baseline irritable bowel syndrome like symptoms (abdominal discomfort and diarrhea) were exacerbated.

**Fig. 1:**
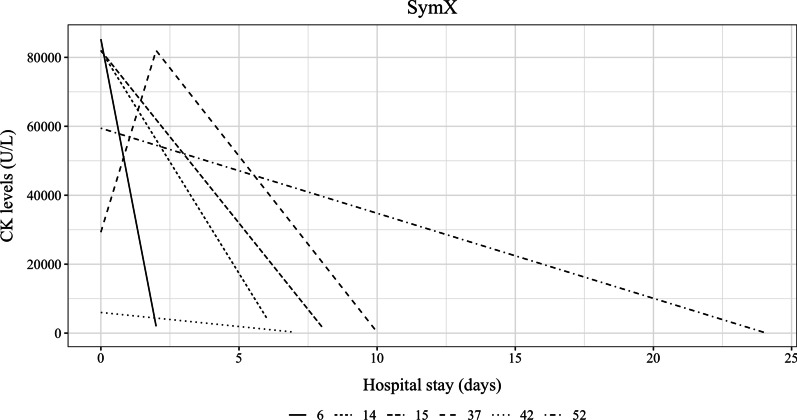
Hospital admissions of six patients with highest CK level at the time of the admission and duration of admission with the discharge CK levels are depicted in Fig. 1 for SymX group

The patient with LCHAD deficiency had 16 acute intercurrent illnesses. One patient with VLCAD deficiency had four acute intercurrent illnesses and the other patient with VLCAD deficiency had 7 acute intercurrent illnesses. One patient with CACT deficiency reported one acute intercurrent illness treated at home. The number of episodes of elevated CK levels ranged from 0 to 19. The average peak CK during acute intercurrent illness or catabolism was 72,783.7 ± 60,708.7 SD U/L (range 246–197,836) (Additional file 1: Table S3). We had details for hospital admission and the CK levels for six patients as depicted in Fig. 1.

### Clinical and biochemical features and treatment outcomes of patients in the AsymX group (*n* = 24)

Twenty-four patients were asymptomatic at the time of diagnosis and were identified by positive NBS (*n* = 9) or by positive family history (*n* = 15) (Table 2). Patients with positive NBS included VLCAD (*n* = 2), LCHAD (*n* = 1), CPT-I (*n* = 1) deficiencies and CTD (*n* = 5). Patients with positive family history included CPT-I (*n* = 7), LCHAD (*n* = 2), VLCAD (*n* = 1), and CACT (n = 1) deficiencies and CTD (n = 4). The average current age was 15.3 ± 16.9 SD years (range 3 months-53 years). The average duration of follow-up was 5.6 ± 3.8 SD years (range 3 months-12 years) in 19 patients, as five asymptomatic patients with CPT-I deficiency were only seen once.

Interestingly, three patients with CTD (mothers of newborn screening positive babies) reported several symptoms retrospectively including fatigue (*n* = 3), muscle pain (*n* = 2), exercise intolerance (*n* = 1), and symptoms of presumed hypoglycemia (e.g., hungry, sweating) (*n* = 1).

Nine patients developed symptoms later including three patients with positive family history and six patients identified by positive NBS. The average lack of time between the diagnosis and symptom onset was 3.1 ± 2.8 SD years (range newborn-8 years). In three patients with positive family history, the symptom onset was during intercurrent illness including two patients with LCHAD deficiency and one patient with CACT deficiency. They presented with rhabdomyolysis, lethargy, hypotonia and hypoglycemia. The average age of symptom onset was 2 ± 3.5 years SD (range newborn-6 years). The average time between the diagnosis and symptom onset was 7.9 ± 13.8 months SD (range newborn-2 years). Six patients identified by positive NBS had subsequent symptom onset including retinopathy in one patient with LCHAD deficiency, rhabdomyolysis in two patients with LCHAD and myalgia in two patients with VLCAD deficiency (Table 2). The average age of symptom onset was 3.2 ± 2.7 SD years (range 1 month-8 years).

Twelve patients had alanine aminotransferase (ALT) (n = 12) and/or aspartate aminotransferase (AST) (n = 9) during regular follow-up appointments. Ten patients (VLCAD *n* = 3; LCHAD *n* = 1 and CACT *n* = 1 deficiencies, and CTD *n* = 5) had normal ALT levels. Nine patients (CTD *n* = 3; LCHAD *n* = 2; VLCAD *n* = 3 and CACT *n* = 1 deficiencies) had normal AST levels.

All patients with CPT-I deficiency (*n* = 8) were asymptomatic. One of the patients with CPT-I deficiency had atrial septal aneurysm with a residual patent foramen ovale and atrial septal defect, residual patent ductus arteriosus, and mild mitral incompetence in echocardiography at age one month. None of the patients with CTD, CACT, LCHAD and VLCAD deficiencies had cardiomyopathy.

**Fig. 2:**
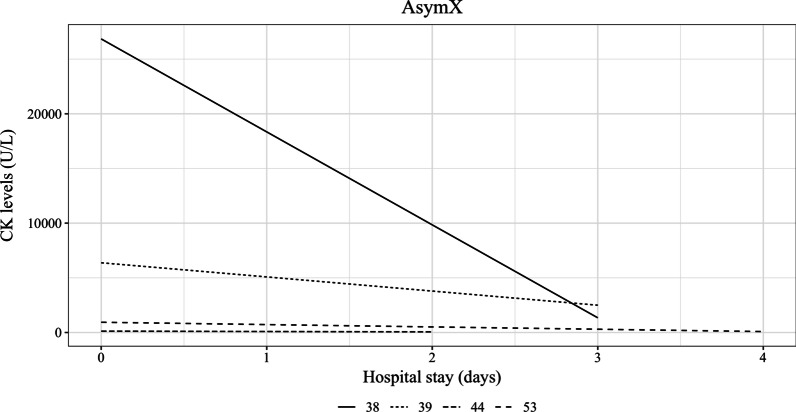
Hospital admissions of four patients with highest CK level at the time of the admission and duration of admission with the discharge CK levels are depicted in Fig. 2 for AsymX group

All patients received illness and emergency management. None of the patients with CPT-I deficiency required any treatments. Eight patients with CTD were treated with carnitine supplementation; one was not compliant with carnitine supplementation. All patients with LCHAD (*n* = 3) and VLCAD (n = 3) deficiencies received long-chain fat restricted diet. The average age of diet initiation was 2.2 ± 4.8 SD months (range newborn-13 months). The average recommended intake of long-chain fat was 18% of total energy intake ± 0.1 SD (range 10–25%). Whereas the average actual intake of long-chain fat was 13% ± 0.1 SD of total energy intake (range 7–26%). The average recommended intake of MCT was 20% of total energy intake ± 0.1 SD (range 13–35%). Whereas the average actual intake of MCT was 19% of total energy intake ± 0.1 SD (range 13–35%). Four patients received walnut oil supplementation (average 1.63 ml, range 0.5–4.0 ml), one patient received flax oil supplementation (0.5 ml), one patient received canola oil supplementation (30 ml), and two patients received cornstarch supplementation (average 0.8 mg/kg/day, range 0.6–0.9 mg/kg/day). The ASympX group demonstrated good adherence to dietary recommendations. 25% (6 out of 24) of the patients were prescribed diet therapy including long chain fat restriction and MCT supplementation. 100% of the patients met their long chain fat goals within 1% of max recommendations or took less long chain fat than prescribed. 83% (5 out of 6) of the patients met their MCT goals. The exception was one child who refused MCT supplementation due to poor palatability. We recommended pre-exercise complex carbohydrates in two patients with VLCAD deficiency to prevent rhabdomyolysis associated with the exercise. Additionally, we recommended pre-exercise complex carbohydrates and MCT oil (in any form) in two patients (VLCAD *n* = 1; and LCHAD *n* = 1 deficiencies) to prevent rhabdomyolysis associated with the exercise (Additional file 1: Table 3). Three patients were compliant with these recommendations including patients with VLCAD (*n* = 2) and LCHAD (*n* = 1) deficiencies and none of these patients had a reduced exercise level. Unfortunately, we are not certain about the compliance of one patient with VLCAD deficiency as the patient is no longer followed by our clinic due to moving out of province. The average number of acute intercurrent illnesses was 6.3 ± 3.3 SD (range 2–10) in patients with LCHAD deficiency. The average number of acute intercurrent illnesses was 4.7 ± 3.8 SD (range 2–9) in patients with VLCAD deficiency. The patient with CACT deficiency reported two acute intercurrent illnesses treated at home. The number of acute episodes of elevated creatine kinase (CK) levels ranged from 0 to 10. The average peak CK during acute intercurrent illness or catabolism was 22,842.5 ± 32,740.3 U/L SD (range 386–70,000) (Additional file 1: Table 3). Details for hospital admission and the CK levels were available for four patients and depicted in Fig. 2.

### Comparison of patients in the SymX group and AsymX group

The symptom frequency for each group is summarized in Table 4. We excluded three patients who were diagnosed due to their positive family history but reported symptoms when they were asked for more information (Table 2). Statistically significant association was found for rhabdomyolysis, and hypoglycemia in the SymX group compared to AsymX group.

The total number of hospital admissions was 16 in one patient with LCHAD deficiency in the SymX group (*n* = 1), whereas the total number of hospital admissions was 12 in three LCHAD patients in the AsymX group (*n* = 3) (Individual admissions: 6, 4, 2) (*p* = 0.500, Wilcoxon rank-sum). The total number of hospital admissions was seven in two patients with VLCAD deficiency in the SymX group (*n* = 2) (individual admissions: 3, 4), whereas the total number of hospital admissions were five in three VLCAD patients in the AsymX group (*n* = 3) (individual admissions: 1, 1, 3) (*p* = 0.224, Wilcoxon rank-sum). The total number of hospital admissions was seven in five patients with CPT-I deficiency in the SymX group (*n* = 5) (individual admissions: 5, 0, 0, 1, 1), whereas none of the patients with CPT-I deficiency in the AsymX group (*n* = 8) (*p* = 0.0225, Wilcoxon rank-sum) had any hospital admissions. A higher number of hospital admissions, longer duration of hospital admissions and higher CK levels were observed in the SymX group, even though the SymX group was only 37% of the study cohort.

## Discussion

We report 38 patients with seven different mitochondrial long-chain fatty acid oxidation and carnitine metabolism defects in our retrospective study from a single metabolic genetics center at our institution. The two most common defects were CPT-I deficiency (27%) and CTD (24%). Interestingly, 38% of the patients with CPT-I deficiency were identified symptomatically, whereas none of the patients with CTD were diagnosed symptomatically but identified due to positive NBS of CTD in their children. Asymptomatic diagnosis and application of treatments appears to decrease the number and duration of hospital admissions as well as peak CK levels. Our study reports the genetic landscape of mitochondrial long-chain fatty acid oxidation and carnitine metabolism defects in pediatric and adult patients from a single center.

The prevalence of maternal CTD was about 1 in 33,000 in a study from Taiwan [24]. In 91 families with abnormal NBS for CTD, 14 mothers were identified with two variants in *SLC22A5*. Eleven of those mothers were asymptomatic and their age ranged from 21 to 43 years at the time of the publication. Fatigability was reported in three of those mothers (21%) [25]. Interestingly, in our study 33% of the mothers reported symptoms at the time of their identification due to the positive NBS of CTD in their children. It is possible that more mothers with maternal CTD may have symptoms and their diagnosis may improve their quality of life and prevent deaths secondary to cardiomyopathy.

The prevalence of CPT-I deficiency in live newborns in three Canadian Territories were reported in 2010. The study genotyped all newborns born in Yukon, Northwest Territories and Nunavut for the p.Pro479Leu variant in *CPT1A* in 2006. The homozygosity rate was 0%, 3%, and 64% respectively in those Canadian Territories [26]. The prevalence of hypoglycemia in term newborns from the Kivalliq region of Nunavut was 22% in newborns with homozygous p.Pro479Leu variant in *CPT1A* [27]. Interestingly, 80% of the SymX patients with CPT-I deficiency had hypoglycemia in our patient cohort. None of the individuals with AsymX CPT-I deficiency reported any symptoms after the genotypic diagnosis. It is important to genotype symptomatic individuals, as asymptomatic individuals do not report any symptoms to the best of our knowledge. If there is a positive family history for symptomatic CPT-I deficiency, newborns in that family can be genotyped to prevent symptoms by application of illness management and emergency letters.

Trauma exposure and post-traumatic stress disorder like behavior in mice were studied using plasma and cerebellum metabolomics analysis. Investigators identified that medium and long chain acylcarnitines were elevated including C8, C12, C14, C16, C18:1 and C18. Additionally, free carnitine was reduced [28]. Interestingly, five adult patients had either suspected or diagnosed mental health condition in our study. Depression was reported in one patient with LCHAD, one patient with CPT-II deficiencies and one patient with CTD. Depression and anxiety were reported in one patient with CPT-I deficiency. Attention deficit disorder was reported in one patient with MAD deficiency. It is important to monitor abnormalities in acylcarnitine and carnitine levels in these patients with mitochondrial long-chain fatty acid oxidation and carnitine metabolism defects. This may increase our understanding of mental health conditions and help formulate better treatments.

Carnitine is a cofactor that transports long-chain fatty acids across the inner mitochondrial membrane for beta oxidation to provide energy to the skeletal and cardiac muscle. Energy demands of skeletal and cardiac muscle strongly require beta oxidation. Approximately 98% of the body’s carnitine is located within cardiac and skeletal muscle tissues. Orally taken carnitine is transported to skeletal and cardiac muscle via carnitine transporters. The half-life of a single dose (2 g) of oral l-carnitine is 60.3 ± 15 min and requires frequent oral intake to maintain carnitine levels. Oral l-carnitine intake of 2 g twice a day increases muscle carnitine by 21% of baseline. Carnitine supplementation increases plasma carnitine levels in individuals with CTD but does not normalize completely. High dose carnitine supplementation (up to 200–250 mg/kg/day) is usually required in individuals with CTD. It has been shown that blood and urine carnitine levels are increased on l-carnitine supplementation. However, there was no measurable increase in intracellular carnitine concentrations. Plasma carnitine levels on oral l-carnitine supplementation do not reflect the tissue carnitine levels. Especially during physical activity, energy demands of skeletal and cardiac muscle increase. It has been shown that during exercise there is an impaired beta oxidation capacity in individuals with CTD which is partially restored by carnitine supplementation [29, 30]. Unfortunately, high dose carnitine supplementation results in a fishy odor to the breath, sweat and urine. The frequent dosing and fishy body odor are major limiting factors for treatment compliance. There are unfortunately sudden deaths in childhood, adolescents, and adults with CTD. The sudden death is attributed to compliance problems, but it has not been proven to be correct. There are no extensive investigations to understand the mechanisms of sudden death in patients with CTD as well as how to prevent this devastating complication. It is timely to think about other treatments in addition to carnitine supplementation such as medium chain triglyceride supplementation or anaplerotic agent such as triheptanoin.

In nine patients with VLCAD deficiency, peripheral blood cells (including natural killer cells, B lymphocytes, T lymphocytes, dendritic cells, hematopoietic stem cells, and hematopoietic progenitor cells) were used to measure oxygen consumption rate and fatty acid oxidation rate. Patients with VLCAD deficiency showed impaired oxidation capacity and reduced total energy production. The supplementation of long-chain fatty acids resulted in the accumulation of long-chain fatty acid in CD8 + T cells, leading to impairment of mitochondrial functions, and decrease in the fatty acid metabolism in these cells [31]. It was previously reported that lymphocyte, monocyte, macrophage, and neutrophil functions were suppressed by long chain fats [32, 33]. The C8:0 supplementation did not result in an increase in oxygen consumption rate in VLCAD patient cells. The authors raised concerns of the efficacy of MCT supplementation in VLCAD deficiency [31]. Additionally, a VLCAD knock-out mouse model infected by influenza showed abnormalities in tissue specific acylcarnitine profiles compared to wild-type [34]. It seems that our patient cohort with mitochondrial long-chain fatty acid oxidation and some of the carnitine metabolism defects have had increased infection frequency resulting in metabolic decompensation and several hospital admissions. This is likely secondary to the energy deficiency and accumulation of long chain fatty acids affecting the immune system functions.

It is important to discuss the improved outcomes in patients who were diagnosed prenatally by positive family history or neonatally by NBS. Both early diagnostic processes are crucial to prevent morbidity and mortality in patients with mitochondrial fatty acid oxidation defects. There are several reports that NBS prevented disease related complications and improved outcomes in patients [4, 8, 12, 35–40]. A recent review article by Marsden et al. summarized outcomes of expanded NBS worldwide. There were several advantages including: 1) there was an increase in the incidence of fatty acid oxidation defects; 2) more patients were identified even if they were symptomatic prior to NBS results; 3) there were improvements in clinical outcomes; 4) there was a decrease in mortality rate; 4) there were less disease related complications due to early identification and treatment of patients with fatty acid oxidation defects identified by NBS such as cardiomyopathy and hypoglycemia; 5) there were improvements in the neurodevelopmental outcomes [41]. In our study, we compared several outcome measures between SymX group and AsymX group. Rhabdomyolysis and hypoglycemia were significantly lower in the AsymX group. None of the patients had myopathy, cardiac arrhythmia, cardiomyopathy, seizures or peripheral neuropathy in the AsymX group in our study.

To report genotype–phenotype correlations for mitochondrial long-chain fatty acid oxidation disorders, we reviewed the literature. Lim et al., [42] reported that the patients with LCHAD deficiency who had either homozygous or heterozygous p.Glu474Gln variant in *HADHA* presented with cardiomyopathy, hypoglycemia, retinopathy, rhabdomyolysis, and peripheral neuropathy [42]. The authors did not report their newborn screening status but compared this common genotype with other genotypes. In our study cohort, all patients with LCHAD deficiency had the common variant and their phenotype was similar to the patients reported in the literature for this genotype. It seems that despite identification by NBS or later in life symptomatically, they present with similar phenotypes. The p.Val283Ala in *ACADVL* was reported in about 10% of newborns identified by NBS by Miller et al. [38]. Interestingly, in our study, only one of the five patients with VLCAD deficiency was heterozygous for this variant who was identified by NBS. Our five patients with VLCAD deficiency were compound heterozygous for eight different variants (75% missense variants). The different genotypes in SymX and AsymX groups for VLCAD deficiency presented with similar phenotypes. The p.Ser113Leu variant in *CPT2* is the common variant for CPT-II deficiency. This genotype is associated with the mild adult-onset phenotype, where most patients were homozygous for this variant [43, 44]. We had two patients with CPT-II deficiency who are heterozygous for this common variant and with a similar phenotype to the patients reported in the literature. Previous reports of large number of patients with CTD did not provide clear genotype–phenotype correlation [3, 7, 25]. We had eight patients with CTD who had nine different variants in our study cohort. We were not able to drove conclusions for genotype–phenotype correlations for those patients with CTD in our study. So far less than 30 patients with CACT deficiency were reported in the literature. There were two patients, 10 years and 16 months of age at the time of the publications who were compound heterozygous for the c.326 + 1delG variant in *SLC25A20*. Their phenotype was similar to our patient’s phenotype [5]. Our sibling pair with CACT deficiency was compound heterozygous for the known p.Asn300Glnfs*24 variant in *SLC25A20.* Interestingly, a patient with CACT deficiency who was homozygous for the same variant had a neonatal onset hypoglycemia but had a milder phenotype and was 9 years old at the time the publication [45]. It appears that this patient and our patients’ phenotypes are similar.

Our study had several limitations including: 1) It is a retrospective cohort study consisting of a chart review of patients who had been diagnosed with mitochondrial long-chain fatty acid oxidation and carnitine metabolism defects at our institution; 2) There were no single evaluation pathway for monitoring of these patients to assess outcomes in detail and systematically; 3) Some of the patients in the AsymX group had no clinical features during the last assessment. It may be still too early to see any symptoms; 4) Our data includes different treatment regimens, and different times for the initiation of treatment and does not allow us to compare the groups, genotypes and treatment outcomes statistically. Despite these limitations, the number of patients from a single metabolic genetics center and their outcomes are valuable and add additional knowledge regarding mitochondrial long-chain fatty acid oxidation and carnitine metabolism defects.

In conclusion, the prevalence of mitochondrial long-chain fatty acid oxidation and carnitine defects was 4.75% in our clinic. Asymptomatic diagnosis is important to improve quality of life and decrease acute complications.

## Supplementary Information

**Additional file 1. Table S1**: Newborn screening results are summarized in Table S1. **Table S2**. All variants and their ACMG classification are summarized in Table S2. **Table S3**: Biochemical features, illness management, and number of lifetime hospital admissions are summarized in Table S3.

## Data Availability

All data generated or analysed during this study are included in this published article and its supplementary information files.
