# Supplementary material for: Outcomes of mitochondrial long chain fatty acid oxidation and carnitine defects from a single center metabolic genetics clinic
Source: Orphanet J Rare Dis. 2022 Sep 15;17:360. doi: 10.1186/s13023-022-02512-5 (PMC9479237; doi:10.1186/s13023-022-02512-5)
Supplement: Supplementary file 1 — Additional file 1. Table S1: Newborn screening results are summarized in Table S1. Table S2. All variants and their ACMG classification are summarized in Table S2. Table S3: Biochemical features, illness management, and number of lifetime hospital admissions are summarized in Table S3. [file 13023_2022_2512_MOESM1_ESM.docx]

**Supplemetal Table 1:** Newborn screening results are summarized in Supplemental Table 1.

| **Patient number/StudyID/diagnosis** | **NBS Free Carnitine and Acylcarnitine** |
| --- | --- |
| 6/12/CPT-I | C0=85.15 |
| 9/18/CUD | C0=10.01 |
| 11/24/CUD | C0=2.50, (C0+C2+C3+C16+C18:1)/Cit=0.372 |
| 12/25/CUD | C0=2.55, (C0+C2+C3+C16+C18:1)/Cit=0.397 |
| 15/28/CUD | C0=8.37 |
| 17/36/CUD | C0=6.66 |
| 18/38/LCHAD | C16OH=1.22, C18OH=1.117, C18:1OH=0.97, C16OH/C16=0.379 |
| 19/39/LCHAD | C16OH=0.88, C18OH=0.95, C18:1OH=0.74, C16OH/C16=0.299 |
| 20/53/LCHAD | C16OH=0.13, C18OH=0.25, C18:1OH=0.13, C16OH/C16=0.078 |
| 21/40/VLCAD | C14:1=3.71, C14:2=0.35, C14:1/C16=0.69 C14:1/C2=0.151 |
| 22/43/VLCAD | C14=3.79, C14:1=4.55, C14:2=0.29, C16:1=1.46, C14:1/C16=0.521 |
| 23/44/VLCAD | C12=0.3, C14:1=0.29, C14:2=0.04, C14:1/C2=0.011 |
| 11/52/VLCAD | C12=0.66, C14=4.45, C14:1=4.70, C14:2=0.25, C16:1=1.81, C14:1/C16=0.501, C14:1/C2=0.392 |

**Reference ranges:** NBS C0 >4; C14 >0.045; C14:1 > 0.06; C14:1/C2 >0.03; C14:1/C16 >0.03; C16OH >0.09; C16OH/16 >0.022

**Abbreviations:** AsymX group= patients identified in neonatal period or due to positive family history; CPT-I=carnitine palmitoyltransferase I; CTD=carnitine transporter; LCHAD=long-chain 3-hydroxyacyl-CoA dehydrogenase; NBS= newborn screening; VLCAD=very long-chain acyl-CoA dehydrogenase

**Supplemental Table 2.** All variants and their ACMG classification are summarized in Supplemental Table 2.

| **Gene**  **(NM#)** | **Variant**  **(references)** | **SIFT** | **MuTaster** | **Polyphen2 (HumVar)** | **Conservation in species (aminos)** | **gmAD allele count in allele #** | **Variant Classification** |
| --- | --- | --- | --- | --- | --- | --- | --- |
| ***CPT1 (NM_001031847.2)*** | C.1436C>T (p.Pro479Leu)  (Bennett et al., 2005; Greenberg et al., 2009) | Deleterious | Disease Causing | Probably Damaging | 7 out of 9 | 9 in 282900 | Pathogenic (PS3, PM2, PP1, PP2, PP3, PP5) |
| ***CPT2 (NM_000098.2)*** | c.298delG (p.Val100LeufsX30)* | NA | Disease Causing | NA | NA | NA | Pathogenic (PVS1, PM2, PP3) |
|  | c.338C>T (p.Ser113Leu)  (Kaufmann et al., 1997; Handig et al., 1996; Corti et al., 2008) | Deleterious | Disease Causing | Probably Damaging | 9 out of 11 | 393 in 282834 | Pathogenic (PVS1, PS3, PS4, PM2, PM3, PP1, PP2, PP3, PP5) |
|  | c.341-2621_1121del* | NA | NA | NA | NA | NA | Likely Pathogenic (PVS1, PM2) |
| ***SLC22A5 (NM_003060.3)*** | c.769C>T (p.Arg257Trp)  (Frigeni et al., 2017; Li et al., 2010) | Deleterious | Disease Causing | Probably Damaging | 7 out of 11 | 12 in 282878 | Likely Pathogenic (PS3, PM2, PP2) |
|  | c.845G>A (p.Arg282Gln)  (Amat et al., 2006; Frigeni et al., 2017; Li et al., 2010) | Deleterious | Disease Causing | possibly damaging | 7 out of 11 | NA | Pathogenic (PS3, PM1, PM2, PP1, PP2, PP3) |
|  | c.248G>T  (p.Arg83Leu)  (Amat et al., 2006; Flippo et al., 2011; Li et al., 2010) | Deleterious | Disease Causing | Probably Damaging | 6 out of 11 | 52 in 193890 | Pathogenic (PS3, PM1, PM2, PP2, PP3, PP5) |
|  | c. 1463G>A (p.Arg488His)    (Amat et al., 2006; Frigeni et al., 2017) | Deleterious | Disease Causing | possibly damaging | 5 out of 11 | 903 in 282826 | VUS (PM5, PP2, BS1, BS2) |
|  | c.424G>T (p.Ala142Ser)  (Li et al., 2010) | tolerated | Disease Causing | benign | 4 out of 11 | 15 in 282902 | Pathogenic (PS3, PM2, PM3, PP2, PP5, BP4) |
|  | c.136C>T (p.Pro46Ser)  (Chen et al., 2013; El-Hattab et al., 2010; Ferdinandusse et al., 2019; Flippo et al., 2011; Li et al., 2011; Schimmenti et al., 2010) | Deleterious | Disease Causing | Probably Damaging | 6 out of 11 | 118 in 275568 | Pathogenic (PS3, PM1, PM2, PM5, PP2, PP3, PP5) |
|  | c.364G>T (p.Asp122Tyr)  (Li et al., 2011; Toh et al., 2011) | Deleterious | Disease Causing | Probably Damaging | 4 out of 11 | 106 in 271794 | Pathogenic (PS3, PM2, PM5, PP2, PP3, PP5) |
|  | c.505C>T (p.Arg169Trp)  (Wang et al., 2000; Li et al., 2010; Lamhonwah et al., 2002; Frigeni et al., 2017; Burwinkel et al., 1999) | Deleterious | Disease Causing | Probably Damaging | 7 out of 11 | 1 in 251448 | Pathogenic (PS3, PM1, PM2, PM5, PP2, PP3, PP5) |
|  | c.1324GC_1325AT (p.Ala442Ile)  (Frigeni et al., 2017; Li et al., 2010) | Deleterious | NA | Probably Damaging | NA | NA | Pathogenic (PS3, PM1, PM2, PM3, PP2, PP3, PP5) |
| ***HADHA (no NM# in the result)*** | c.1528G>C (p.Glu474Gln)  (Tabor et al., 2014; Spiekerkoetter et al., 2004; Sims et al., 1995; Olpin et al., 2005; Karall et al., 2015; IJIst et al., 1994) | Deleterious | Disease Causing | Probably Damaging | 10 out of 11 | 366 in 282830 | Pathogenic (PS3, PS4, PP1, PP2, PP3, PP5) |
| ***ACADVL (NM_000018.2)*** | c.1375dupC (p.Arg459Profsx4)  (Miller et al., 2015) | NA | NA | NA | NA | NA | Pathogenic (PVS1, PM2, PP5) |
|  | c.1700G>A (p.Arg567Gln)  (Ficicioglu et al., 2010; Schiff et al., 2013) | Deleterious | Disease Causing | Possibly damaging | 9 out of 11 | NA | Likely Pathogenic (PS3, PM2, PP2, PP3, PP5) |
|  | c.605T>A (p.Leu202His)  (Rovelli et al., 2019) | Deleterious | Disease Causing | Probably Damaging | 8 out of 11 | NA | Likely Pathogenic (PM1, PM2, PM5, PP2, PP3) |
|  | c.1182+1G>A  (Vallejo et al., 2021; Strauss et al., 1995; Schiff et al., 2013; Pena et al., 2016) | NA | NA | NA | NA | NA | Pathogenic (PVS1, PS3, PM2, PP5) |
|  | c.848T>C (p.Val283Ala)  (Andresen et al., 1999; Goetzman et al., 2007; Hoffmann et al., 2012; Liebig et al., 2006; Marsden et al., 2021; Mathur et al., 1999; Schiff et al., 2013) | Deleterious | Disease Causing | benign | 5 out of 11 | 2 in 251458 | Pathogenic (PS3, PM1, PM2, PP2, PP5) |
|  | c.779C>T (p.Thr260Met)  (Tabor et al., 2014; Mathur et al., 1999; Liebig et al., 2006; Hoffmann et al., 2012; Goetzman et al., 2007; Andresen et al., 1996) | Deleterious | Disease Causing | Probably Damaging | 9 out of 11 | NA | Pathogenic (PS3, PM1, PM2, PP2, PP3, PP5) |
|  | c.1733T>C (p.Met578Thr)  (Miller et al., 2015) | Deleterious | Disease Causing | possibly damaging | 8 out of 11 | NA | VUS (PM2, PP2) |
|  | c.1406G>A p.(Arg469Gln)  (Andresen et al., 1999) | Deleterious | Disease Causing | Probably damaging | 8 out of 9 | NA | Likely Pathogenic (PM1, PM2, PM5, PP3, PP5) |
| ***SLC25A20 (NM_000387.5)*** | c.897dupC (p.Asn300Glnfs*24)  (Huizing et al., 1998; Wang et al., 2011) | NA | NA | NA | NA | 1 in 250746 | Pathogenic (PS3, PM2, PM4, PP1, PP5) |
|  | c.269T>G (p.Phe90Cys)* | Deleterious | Disease Causing | Probably Damaging | 11 out of 11 | 1 in 250358 | VUS (PM2, PP1, PP3) |
|  | c.326+1delG (IVS3+1delG)  (Yang et al., 2001; Korman et al., 1997; Iacobazzi et al., 2004; Hsu et al., 2001) | NA | NA | NA | NA | 9 in 249670 | Pathogenic (PVS1, PS3, PM2, PP1, PP2, PP5) |
|  | c.10C>T (p.Gln4X)* | NA | Disease Causing | NA | NA | NA | Pathogenic (PVS1, PM2, PP5) |
| ***ETFDH (NM_004453.2)*** | c.524G>A (p.Arg175His)  (Wang et al., 2011; Yotsumoto et al., 2008) | Deleterious | Disease Causing | Probably Damaging | 8 out of 11 | 4 in 251342 | Likely Pathogenic (PM2, PM3, PM5, PP1, PP2, PP3, PP5) |
|  | c. 1001T>C (p.Leu334Pro)  (Cornelius et al., 2012; Goodman et al., 2002; Olsen et al., 2007) | Deleterious | Disease Causing | Probably Damaging | 7 out of 11 | 8 in 282776 | Likely Pathogenic (PM2, PP2, PP3, PP5) |

* Indicates a novel variant.

**Supplemental Table 3:** Biochemical features, illness management, exercise plan and number of lifetime hospital admissions are summarized in Supplemental Table 3.

|  | **Patient#/patient ID/Diagnosis/age at diagnosis/current age** | **CK range during illness**  **Min max** | **Illness management/ formula/ exercise plan** | **Number of hospital admissions** |
| --- | --- | --- | --- | --- |
| **SymX group** | 1/1/CPT1-I def/2yrs/26yrs | 203 | Consume carbohydrate (20-30g) every 3hrs, watch for signs of low blood glucose, ER if unable to tolerate food or drink or vomit more than 2-3 times | 5 |
|  | 2/3/CPT1-I def/neonatal/3yrs | None | ER if unable to tolerate food or drink or if display symptoms of fever, lethargy, vomiting | 0 |
|  | 3/7/CPT-I def/2mo/3yrs | None | ER if unable to tolerate food or drink or if display symptoms of fever, lethargy, vomiting | 0 |
|  | 4/10/CPT-I def/neonatal/3yrs | NR | ER if unable to tolerate food or drink or if display symptoms of fever, lethargy, vomiting | 1 |
|  | 5/49/CPT-I def/5ys/22yrs | NR | ER if unable to tolerate food or drink or if display symptoms of fever, lethargy, vomiting | 1 |
|  | 6/6/CPT-II def/35yrs/51yrs | 85280 – 197836 | ER if unable to tolerate food or drink or if display symptoms of rhabdomyolysis/carnitine 750mg IV four times per day/ Pre-exercise consume carbohydrate snack, coompliant, moderate exercise level | 3 |
|  | 7/14/CPT-II def/15yrs/24yrs | 4115 – 82000 | 3-4L fluid, minimize long-chain fat intake, ER if unable to tolerate food or drink or if display symptoms of rhabdomyolysis/42g MCT procal/ Consume 1 tbsp (0.3g/kg) MCT oil pre-exercise, 250 mL powerade or juice (15 g carbohydrate) pre-exercise for light-moderate exercise, within 45 minutes post-exercise consume 100-200 kcal snack (20g CHO, 7g protein), consume 1.5 tbsp MCT oil (21g MCT) for 3-days post-exercise, non-compliant with MCT goal, high exercise level. | 12 |
|  | 8/15/CPT-II def/35yrs/36yrs | 57712 – 82000 | Decrease max fast to 6 hrs, minimize long-chain fat, ER if unable to tolerate food or drink or if display symptoms of rhabdomyolysis/60 mL skim milk+23g solcarb+12g MCT procal/pre-exercise consume 0.2g/kg MCT (13-14g)+complex carbohydrate, during exercise consume complex carbohydrate every 30 min+fluid throughout, max 1.5 hours duration,  poor compliance as does not always consume complex carbohydrate and is not taking MCT with exercise, high exercise level | 2 |
|  | 9/37/LCHAD def/47yrs/55yrs | 2039 – 82000 | Increase formula to 720mL per day, minimuze long-chain fat intake/200g Solcarb+1000mL water/ Pre-exercise consume half a bottle of Betaquick (22.5g MCT, 1g LCF) or 375 mL of Lipistart recipe (23g MCT, 3g LCF), poor compliance as does not take MCT, low exercise level | 16 |
|  | 10/42/VLCAD def/10mo/25yrs | 496 – 6000 | ER if unable to tolerate food or drink or if display symptoms such as lethargy, fever, vomiting/ Pre-exercise consume 0.15-0.2g/kg MCT oil, overall compliant though pre-exercise snack does not always include MCT, high exercise level | 3 |
|  | 11/52/VLCAD def/neonatal/7mo | 4800 – 59447 | No home management | 4 |
|  | 12/45/CACT def/neonatal/3yrs | None | Maximum fast 12hrs, ER if unable to tolerate food or drink or if display symptoms of fever, lethargy, vomiting/32g MCT procal+120mL skim milk+25% carbohydrate solution+1300mL water | 0 |
|  | 13/47/CACT def/neonatal/deceased | NR | None | 1 |
|  | 14/48/MAD def/4yrs/26yrs | NR | Continue carnitine supplementation, decrease protein intake to 45g, max fast 8-10 hrs/320 g solcarb+1000mL water/ Pre-exercise consume ~74g Solcarb or up to 0.8g/kg carbohydrate, overall compliant though takes additional fitness supplements, high exercise level | 3 |
| **AsymX group** | 1/2/CPT-I def/neonatal/3yrs | None | ER if unable to tolerate food or drink or if display symptoms of fever, lethargy, vomiting | 0 |
|  | 2/5/CPT-I def/6yrs/26yrs | None | Consume carbohydrate (20-30g) every 3hrs, watch for signs of low blood glucose, ER if unable to tolerate food or drink or vomit more than 2-3 times | 0 |
|  | 3/8/CPT-I def/45yrs/53yrs | None | Consume carbohydrate (20-30g) every 3hrs, watch for signs of low blood glucose, ER if unable to tolerate food or drink or vomit more than 2-3 times | 0 |
|  | 4/9/CPT-I def/neonatal/6yrs | None | ER if unable to tolerate food or drink or if display symptoms of fever, lethargy, vomiting | 0 |
|  | 5/11/CPT-I def/39yrs/48yrs | None | Consume carbohydrate (20-30g) every 3hrs, watch for signs of low blood glucose, ER if unable to tolerate food or drink or vomit more than 2-3 times | 0 |
|  | 6/12/CPT-I def/neonatal/2yrs | None | ER if unable to tolerate food or drink or if display symptoms of fever, lethargy, vomiting | 0 |
|  | 7/50/CPT-I def/2yrs/3yrs | None | ER if unable to tolerate food or drink or if display symptoms of fever, lethargy, vomiting | 0 |
|  | 8/51/CPT-I def/neonatal/5yrs | None | ER if unable to tolerate food or drink or if display symptoms of fever, lethargy, vomiting | 0 |
|  | 9/18/CTD/neonatal/7yrs | None | None | 0 |
|  | 10/19/CTD/32yrs/37yrs | None | None | 0 |
|  | 11/24/CTD/neonatal/3yrs | NR | None | 0 |
|  | 12/25/CTD/neonatal/3mo | None | None | 0 |
|  | 13/22/CTD/38yrs/44yrs | None | None | 0 |
|  | 14/23/CTD/28yrs/40yrs | None | None | 0 |
|  | 15/28/CTD/neonatal/6yrs | None | None | 0 |
|  | 16/32/CTD/26yrs/33yrs | None | None | 0 |
|  | 17/36/CTD/neonatal/9yrs | None | None | 0 |
|  | 18/38/LCHAD def/neonatal/6yrs | 11140 – 70000 | 115%-120% kcals, additional 250mL fluids, stop regular prescribed long-chain fat, complete blood work, go to ER when unable to tolerate food or drink or if display symptoms like muscle pain, lethargy, irritability, pallor/90g Lipistart+180g Solcarb+110g MCT Procal+300mL skim milk+water up to 1800mL/ Pre-exercise consume complex carbohydrate snack+5-10mL additional MCT oil, consume Gatorade during exercise, post-exercise consume and carbohydrate snack, compliant, high exercise level. | 6 |
|  | 19/39/LCHAD def/neonatal/3yrs | 345 – 20047 | Stop regular prescribed long-chain fat, 300 mL additional fluid, complete blood work, ER if unable to tolerate food or drink or display symptoms such as muscle pain, lethargy, irritability or pallor/140g Lipistart+32g MCT procal+115g solcarb+600mL skim milk+water up to 1400mL | 4 |
|  | 20/53/LCHAD def/neonatal/7mo | 358 – 937 | Max fast 2 hrs, ER if unable to tolerate formula/125g lipistart+8g MCT procal+12mL solcarb | 2 |
|  | 21/40/VLCAD def/neonatal/7yrs | 153 | Continue MCT as able, ER if unable tolerate food or drink or display symptoms like fever, lethargy, vomiting/30% carbohydrate (Using juice, pop or added sugar)/ Pre-exercise consume carbohydrate snack, compliant, low exercise level. | 1 |
|  | 22/43/VLCAD def/neonatal/12yrs | NR | Continue MCT as able, ER if unable tolerate food or drink or display symptoms like fever, lethargy, vomiting/25% Solcarb solution/ Consume additional 17.5mL MCT oil on high exercise days, consume 25% Solcarb or Gatorade throughut exercise, post-exercise consume 3 carbohydrate:1 protein snack (100-200 kcal) within 45 minutes of end, compliant, moderate level of exercise | 1 |
|  | 23/44/VLCAD def/neonatal/9yrs | 102 – 137 | Inform dietician, continue normal MCT as able/53g solcarb+200mL water, or 265g Solcarb+1000mL water, or 48g solcarb+200mL oral electrolyte solution/ Consume carbohydrate snack pre- and post-exercise, compliance and level of exercise unreported. | 3 |
|  | 24/46/CACT def/4yrs/8yrs | None | Maximum fast 12hrs, ER if unable to tolerate food or drink or if display symptoms of fever, lethargy, vomiting/48g MCT procal+120mL skim milk+25% carbohydrate solution+1150mL water | 0 |

**Abbreviations**: CACT= carnitine acylcarnitine translocase; CPT-I=carnitine palmitoyltransferase I; CPT-II=carnitine palmitoyltransferase II; CTD=carnitine transporter defect; ER=emergency room; g=grams; hrs=hours; IV=intravenous; LCHAD=long-chain 3-hydroxyacyl-CoA dehydrogenase; MAD= multiple acyl-CoA dehydrogenase; max=maximum; MCT=medium chain triglyceride; mL=milliliters; mo=months; NR=not reported; yrs=years; VLCAD=very long-chain acyl-CoA dehydrogenase

**Reference Ranges:** CK male with LCHAD deficiency: 30-350 U/L; CK female with LCHAD deficiency: 30-200 U/L, CK female with VLCAD deficiency: 30-200 U/L; CK female with CACT deficiency: 40-220 U/L; CK patient with CPT-II deficiency: 30-200 U/L; CK patient with MAD deficiency: <250 U/L; CK female with CPT1 deficiency: <200 U/L

**References (listed alphabetically)**

Amat di San Filippo C, Pasquali M, Longo N. Pharmacological rescue of carnitine transport in primary carnitine deficiency. Hum Mutat. 2006 Jun;27(6):513-23.

Andresen BS, Bross P, Vianey-Saban C, Divry P, Zabot MT, Roe CR, Nada MA, Byskov A, Kruse TA, Neve S, Kristiansen K, Knudsen I, Corydon MJ, Gregersen N. Cloning and characterization of human very-long-chain acyl-CoA dehydrogenase cDNA, chromosomal assignment of the gene and identification in four patients of nine different mutations within the VLCAD gene. Hum Mol Genet. 1996 Apr;5(4):461-72.

Andresen BS, Olpin S, Poorthuis BJ, Scholte HR, Vianey-Saban C, Wanders R, Ijlst L, Morris A, Pourfarzam M, Bartlett K, Baumgartner ER, deKlerk JB, Schroeder LD, Corydon TJ, Lund H, Winter V, Bross P, Bolund L, Gregersen N. Clear correlation of genotype with disease phenotype in very-long-chain acyl-CoA dehydrogenase deficiency. Am J Hum Genet. 1999 Feb;64(2):479-94. (22)

Bennett MJ, Santani AB. Carnitine Palmitoyltransferase 1A Deficiency. 2005 Jul 27 [updated 2016 Mar 17]. In: Adam MP, Ardinger HH, Pagon RA, Wallace SE, Bean LJH, Gripp KW, Mirzaa GM, Amemiya A, editors. GeneReviews [Internet]. Seattle (WA): University of Washington, Seattle; 1993–2022.

Burwinkel B, Kreuder J, Schweitzer S, Vorgerd M, Gempel K, Gerbitz KD, Kilimann MW. Carnitine transporter OCTN2 mutations in systemic primary carnitine deficiency: a novel Arg169Gln mutation and a recurrent Arg282ter mutation associated with an unconventional splicing abnormality. Biochem Biophys Res Commun. 1999 Aug 2;261(2):484-7.

Chen YC, Chien YH, Chen PW, Leung-Sang Tang N, Chiu PC, Hwu WL, Lee NC. Carnitine uptake defect (primary carnitine deficiency): risk in genotype-phenotype correlation. Hum Mutat. 2013 Apr;34(4):655.

Cornelius N, Frerman FE, Corydon TJ, Palmfeldt J, Bross P, Gregersen N, Olsen RK. Molecular mechanisms of riboflavin responsiveness in patients with ETF-QO variations and multiple acyl-CoA dehydrogenation deficiency. Hum Mol Genet. 2012 Aug 1;21(15):3435-48.

Corti S, Bordoni A, Ronchi D, Musumeci O, Aguennouz M, Toscano A, Lamperti C, Bresolin N, Comi GP. Clinical features and new molecular findings in Carnitine Palmitoyltransferase II (CPT II) deficiency. J Neurol Sci. 2008;266(1-2):97-103.

El-Hattab AW, Li FY, Shen J, Powell BR, Bawle EV, Adams DJ, Wahl E, Kobori JA, Graham B, Scaglia F, Wong LJ. Maternal systemic primary carnitine deficiency uncovered by newborn screening: clinical, biochemical, and molecular aspects. Genet Med. 2010 Jan;12(1):19-24.

Ferdinandusse, S., Te Brinke, H., Ruiter, J., Haasjes, J., Oostheim, W., van Lenthe, H., IJlst, L., Ebberink, M. S., Wanders, R., Vaz, F. M., & Waterham, H. R. (2019). A mutation creating an upstream translation initiation codon in SLC22A5 5'UTR is a frequent cause of primary carnitine deficiency. Human mutation, 40(10), 1899–1904.

Ficicioglu C, Coughlin CR 2nd, Bennett MJ, Yudkoff M. Very long-chain acyl-CoA dehydrogenase deficiency in a patient with normal newborn screening by tandem mass spectrometry. J Pediatr. 2010 Mar;156(3):492-4.

Filippo CA, Ardon O, Longo N. Glycosylation of the OCTN2 carnitine transporter: study of natural mutations identified in patients with primary carnitine deficiency. Biochim Biophys Acta. 2011 Mar;1812(3):312-20.

Frigeni M, Balakrishnan B, Yin X, Calderon FRO, Mao R, Pasquali M, Longo N. Functional and molecular studies in primary carnitine deficiency. Hum Mutat. 2017 Dec;38(12):1684-1699.

Goetzman ES, Wang Y, He M, Mohsen AW, Ninness BK, Vockley J. Expression and characterization of mutations in human very long-chain acyl-CoA dehydrogenase using a prokaryotic system. Mol Genet Metab. 2007 Jun;91(2):138-47.

Goodman SI, Binard RJ, Woontner MR, Frerman FE. Glutaric acidemia type II: gene structure and mutations of the electron transfer flavoprotein:ubiquinone oxidoreductase (ETF:QO) gene. Mol Genet Metab. 2002 Sep-Oct;77(1-2):86-90.

Greenberg CR, Dilling LA, Thompson GR, Seargeant LE, Haworth JC, Phillips S, Chan A, Vallance HD, Waters PJ, Sinclair G, Lillquist Y, Wanders RJ, Olpin SE. The paradox of the carnitine palmitoyltransferase type Ia P479L variant in Canadian Aboriginal populations. Mol Genet Metab. 2009 Apr;96(4):201-7.

Handig I, Dams E, Taroni F, Van Laere S, de Barsy T, Willems P J. Inheritance of the S113L mutation within an inbred family with carnitine palmitoyltransferase enzyme deficiency. Hum Genet. 1996;97(3):291-293.

Hoffmann L, Haussmann U, Mueller M, Spiekerkoetter U. VLCAD enzyme activity determinations in newborns identified by screening: a valuable tool for risk assessment. J Inherit Metab Dis. 2012 Mar;35(2):269-77.

Hsu BY, Iacobazzi V, Wang Z, Harvie H, Chalmers RA, Saudubray JM, Palmieri F, Ganguly A, Stanley CA. Aberrant mRNA splicing associated with coding region mutations in children with carnitine-acylcarnitine translocase deficiency. Mol Genet Metab. 2001;74(1-2):248-255.

Huizing M, Wendel U, Ruitenbeek W, Iacobazzi V, IJlst L, Veenhuizen P, Savelkoul P, van den Heuvel LP, Smeitink JA, Wanders RJ, Trijbels JM, Palmieri F. Carnitine-acylcarnitine carrier deficiency: identification of the molecular defect in a patient. J Inherit Metab Dis. 1998;21(3):262-267.

Iacobazzi V, Invernizzi F, Baratta S, Pons R, Chung W, Garavaglia B, Dionisi-Vici C, Ribes A, Parini R, Huertas MD, Roldan S, Lauria G, Palmieri F, Taroni F. Molecular and functional analysis of SLC25A20 mutations causing carnitine-acylcarnitine translocase deficiency. Hum Mutat. 2004 Oct;24(4):312-20.

IJlst L, Wanders RJ, Ushikubo S, Kamijo T, Hashimoto T. Molecular basis of long-chain 3-hydroxyacyl-CoA dehydrogenase deficiency: identification of the major disease-causing mutation in the alpha-subunit of the mitochondrial trifunctional protein. Biochim Biophys Acta. 1994 Dec 8;1215(3):347-50.

Karall D, Brunner-Krainz M, Kogelnig K, Konstantopoulou V, Maier EM, Möslinger D, Plecko B, Sperl W, Volkmar B, Scholl-Bürgi S. Clinical outcome, biochemical and therapeutic follow-up in 14 Austrian patients with Long-Chain 3-Hydroxy Acyl CoA Dehydrogenase Deficiency (LCHADD). Orphanet J Rare Dis. 2015 Feb 22;10:21.

Kaufmann P, el-Schahawi M, DiMauro S. Carnitine palmitoyltransferase II deficiency: diagnosis by molecular analysis of blood. Mol Cell Biochem. 1997;174(1-2):237-239.

Korman SH, Pitt JJ, Boneh A, Dweikat I, Zater M, Meiner V, Gutman A, Brivet M. A novel SLC25A20 splicing mutation in patients of different ethnic origin with neonatally lethal carnitine-acylcarnitine translocase (CACT) deficiency. Mol Genet Metab. 2006 Dec;89(4):332-8.

Lamhonwah AM, Olpin SE, Pollitt RJ, Vianey-Saban C, Divry P, Guffon N, Besley GT, Onizuka R, De Meirleir LJ, Cvitanovic-Sojat L, Baric I, Dionisi-Vici C, Fumic K, Maradin M, Tein I. Novel OCTN2 mutations: no genotype-phenotype correlations: early carnitine therapy prevents cardiomyopathy. Am J Med Genet. 2002 Aug 15;111(3):271-84.

Li FY, El-Hattab AW, Bawle EV, Boles RG, Schmitt ES, Scaglia F, Wong LJ. Molecular spectrum of SLC22A5 (OCTN2) gene mutations detected in 143 subjects evaluated for systemic carnitine deficiency. Hum Mutat. 2010 Aug;31(8):E1632-51. (5,4,11,13,6,9) 19

Liebig M, Schymik I, Mueller M, Wendel U, Mayatepek E, Ruiter J, Strauss AW, Wanders RJ, Spiekerkoetter U. Neonatal screening for very long-chain acyl-coA dehydrogenase deficiency: enzymatic and molecular evaluation of neonates with elevated C14:1-carnitine levels. Pediatrics. 2006 Sep;118(3):1065-9.

Marsden D, Bedrosian CL, Vockley J. Impact of newborn screening on the reported incidence and clinical outcomes associated with medium- and long-chain fatty acid oxidation disorders. Genet Med. 2021 May;23(5):816-829.

Mathur A, Sims HF, Gopalakrishnan D, Gibson B, Rinaldo P, Vockley J, Hug G, Strauss AW. Molecular heterogeneity in very-long-chain acyl-CoA dehydrogenase deficiency causing pediatric cardiomyopathy and sudden death. Circulation. 1999 Mar 16;99(10):1337-43.

Miller MJ, Burrage LC, Gibson JB, Strenk ME, Lose EJ, Bick DP, Elsea SH, Sutton VR, Sun Q, Graham BH, Craigen WJ, Zhang VW, Wong LJ. Recurrent ACADVL molecular findings in individuals with a positive newborn screen for very long chain acyl-coA dehydrogenase (VLCAD) deficiency in the United States. Mol Genet Metab. 2015 Nov;116(3):139-45.

Olpin SE, Clark S, Andresen BS, Bischoff C, Olsen RK, Gregersen N, Chakrapani A, Downing M, Manning NJ, Sharrard M, Bonham JR, Muntoni F, Turnbull DN, Pourfarzam M. Biochemical, clinical and molecular findings in LCHAD and general mitochondrial trifunctional protein deficiency. J Inherit Metab Dis. 2005;28(4):533-44.

Olsen RK, Olpin SE, Andresen BS, Miedzybrodzka ZH, Pourfarzam M, Merinero B, Frerman FE, Beresford MW, Dean JC, Cornelius N, Andersen O, Oldfors A, Holme E, Gregersen N, Turnbull DM, Morris AA. ETFDH mutations as a major cause of riboflavin-responsive multiple acyl-CoA dehydrogenation deficiency. Brain. 2007 Aug;130(Pt 8):2045-54.

Pena LD, van Calcar SC, Hansen J, Edick MJ, Walsh Vockley C, Leslie N, Cameron C, Mohsen AW, Berry SA, Arnold GL, Vockley J; IBEMC. Outcomes and genotype-phenotype correlations in 52 individuals with VLCAD deficiency diagnosed by NBS and enrolled in the IBEM-IS database. Mol Genet Metab. 2016 Aug;118(4):272-81.

Rovelli V, Manzoni F, Viau K, Pasquali M, Longo N. Clinical and biochemical outcome of patients with very long-chain acyl-CoA dehydrogenase deficiency. Mol Genet Metab. 2019 May;127(1):64-73.

Schiff M, Mohsen AW, Karunanidhi A, McCracken E, Yeasted R, Vockley J. Molecular and cellular pathology of very-long-chain acyl-CoA dehydrogenase deficiency. Mol Genet Metab. 2013 May;109(1):21-7.

Schimmenti LA, Crombez EA, Schwahn BC, Heese BA, Wood TC, Schroer RJ, Bentler K, Cederbaum S, Sarafoglou K, McCann M, Rinaldo P, Matern D, di San Filippo CA, Pasquali M, Berry SA, Longo N. Expanded newborn screening identifies maternal primary carnitine deficiency. Mol Genet Metab. 2007 Apr;90(4):441-5.

Sims HF, Brackett JC, Powell CK, Treem WR, Hale DE, Bennett MJ, Gibson B, Shapiro S, Strauss AW. The molecular basis of pediatric long chain 3-hydroxyacyl-CoA dehydrogenase deficiency associated with maternal acute fatty liver of pregnancy. Proc Natl Acad Sci U S A. 1995 Jan 31;92(3):841-5.

Spiekerkoetter U, Khuchua Z, Yue Z, Bennett MJ, Strauss AW. General mitochondrial trifunctional protein (TFP) deficiency as a result of either alpha- or beta-subunit mutations exhibits similar phenotypes because mutations in either subunit alter TFP complex expression and subunit turnover. Pediatr Res. 2004 Feb;55(2):190-6.

Strauss AW, Powell CK, Hale DE, Anderson MM, Ahuja A, Brackett JC, Sims HF. Molecular basis of human mitochondrial very-long-chain acyl-CoA dehydrogenase deficiency causing cardiomyopathy and sudden death in childhood. Proc Natl Acad Sci U S A. 1995 Nov 7;92(23):10496-500.

Tabor HK, Auer PL, Jamal SM, Chong JX, Yu JH, Gordon AS, Graubert TA, O'Donnell CJ, Rich SS, Nickerson DA; NHLBI Exome Sequencing Project, Bamshad MJ. Pathogenic variants for Mendelian and complex traits in exomes of 6,517 European and African Americans: implications for the return of incidental results. Am J Hum Genet. 2014 Aug 7;95(2):183-93.

Toh DS, Murray M, Pern Tan K, Mulay V, Grewal T, Lee EJ, Zhou F. Functional analysis of pharmacogenetic variants of human organic cation/carnitine transporter 2 (hOCTN2) identified in Singaporean populations. Biochem Pharmacol. 2011 Dec 1;82(11):1692-9.

Vallejo AN, Mroczkowski HJ, Michel JJ, Woolford M, Blair HC, Griffin P, McCracken E, Mihalik SJ, Reyes-Mugica M, Vockley J. Pervasive inflammatory activation in patients with deficiency in very-long-chain acyl-coA dehydrogenase (VLCADD). Clin Transl Immunology. 2021 Jun 27;10(6):e1304.

Wang Y, Taroni F, Garavaglia B, Longo N. Functional analysis of mutations in the OCTN2 transporter causing primary carnitine deficiency: lack of genotype-phenotype correlation. Hum Mutat. 2000 Nov;16(5):401-7.

Wang GL, Wang J, Douglas G, Browning M, Hahn S, Ganesh J, Cox S, Aleck K, Schmitt ES, Zhang W, Wong LJ. Expanded molecular features of carnitine acyl-carnitine translocase (CACT) deficiency by comprehensive molecular analysis. Mol Genet Metab. 2011 Aug;103(4):349-57.

Wang ZQ, Chen XJ, Murong SX, Wang N, Wu ZY. Molecular analysis of 51 unrelated pedigrees with late-onset multiple acyl-CoA dehydrogenation deficiency (MADD) in southern China confirmed the most common ETFDH mutation and high carrier frequency of c.250G>A. J Mol Med (Berl). 2011 Jun;89(6):569-76.

Yang BZ, Mallory JM, Roe DS, Brivet M, Strobel GD, Jones KM, Ding JH, Roe CR. Carnitine/acylcarnitine translocase deficiency (neonatal phenotype): successful prenatal and postmortem diagnosis associated with a novel mutation in a single family. Mol Genet Metab. 2001;73(1):64-70.

Yotsumoto Y, Hasegawa Y, Fukuda S, Kobayashi H, Endo M, Fukao T, Yamaguchi S. Clinical and molecular investigations of Japanese cases of glutaric acidemia type 2. Mol Genet Metab. 2008 May;94(1):61-7.
